# Responses of promyelocytic leukemia HL60 cells as an inflammatory cell lineage model to silica microparticles used to coat blood collection tubes

**DOI:** 10.1186/s40729-022-00424-4

**Published:** 2022-05-14

**Authors:** Hideo Masuki, Takashi Uematsu, Hideo Kawabata, Atsushi Sato, Taisuke Watanabe, Tetsuhiro Tsujino, Masayuki Nakamura, Masaya Okubo, Tomoyuki Kawase

**Affiliations:** 1 Tokyo Plastic Dental Society, Kita-Ku, Tokyo, Japan; 2 Division of Oral Bioengineering, Institute of Medicine and Dentistry, Niigata University, Niigata, Japan

**Keywords:** Silica, HL60 cells, Viability, Differentiation, Superoxide dismutase

## Abstract

**Background:**

The preparation of platelet-rich fibrin (PRF) requires glass blood collection tubes, and thus, the shortage or unavailability of such tubes has driven clinicians to search for suitable substitutes, such as silica-coated plastic tubes. However, we have previously demonstrated the cytotoxicity of silica microparticles (MPs) used in plastic tubes to cultured human periosteal cells. To further establish the effects of silica MPs on inflammation, we examined silica MP-induced changes in a human promyelocytic cell model in vitro.

**Methods:**

Human promyelocytic HL60 cells were used either without chemical induction or after differentiation induced using phorbol myristate acetate (PMA) or dimethyl sulfoxide. HL60 cells, osteoblastic MG63, and Balb/c mouse cells were treated with silica MPs, and their surface ultrastructure and numbers were examined using a scanning electron microscope and an automated cell counter, respectively. Differentiation markers, such as acid phosphatase, non-specific esterase, and CD11b, were visualized by cytochemical and immunofluorescent staining, and superoxide dismutase (SOD) activity was quantified.

**Results:**

Regardless of SOD activity, silica cytotoxicity was observed in MG63 and Balb/c cells. At sub-toxic doses, silica MPs slightly or moderately upregulated the differentiation markers of the control, PMA-induced monocytic, and dimethyl sulfoxide-induced granulocytic HL60 cells. Although SOD activity was the highest (*P* < 0.05) in PMA-induced cells, a silica-induced reduction in cell adhesion was observed only in those cells (*P* < 0.05).

**Conclusions:**

Silica MP contamination of PRF preparations can potentially exacerbate inflammation at implantation sites. Consequently, unless biomedical advantages can be identified, silica-coated plastic blood collection tubes should not be routinely used for PRF preparations.

## Introduction

Platelet-rich plasma is used in regenerative therapy in a wide range of fields. In the mid-2000s, platelet-rich fibrin (PRF) was established as a second-generation derivative of platelet-rich plasma, the advantage of which is that it does not require the use of specific anticoagulants, coagulation factors, or preparation skills. However, the preparation of this fibrin matrix requires the use of appropriate glass tubes for coagulation, which represents a serious disadvantage in countries or regions in which glass vacuum tubes are commercially unavailable. Consequently, clinicians have been seeking suitable substitutes, for example, silica-coated plastic tubes. Although glass is more inert and chemically more stable than plastic material, neither manufacturers nor regulatory agencies have officially commented on the reasons for discontinued or reduced production. Instead, it has been suggested that glass products are relatively more fragile, costlier in production and distribution, and bulkier upon disposal than plastic products.

Regardless of the reason, the use of silica-coated plastic tubes, which are produced for laboratory testing, is accepted as the most affordable way to continue routine PRF therapy. Unfortunately, despite the fact that we have previously reported the inappropriate use and cytotoxic effects of silica-coated plastic tubes [1, 2], several vendors still recommend the use of these tubes for PRF preparation. These types of blood collection tubes were originally designed for use in laboratory testing; moreover, almost all manufacturers recommend that they should not be used to produce materials intended for subsequent use in implantation. Nevertheless, some clinicians continue to use them at their discretion, irrespective of the potential hazards posed by these products.

Although widely found in a range of routinely used products and indispensable for our lives, silica has been identified as a causal factor for certain disorders, including lung silicosis, a type of pulmonary fibrosis caused by the inhalation of minute particles of silica [3, 4]. Silicosis primarily affects workers exposed to silica dust, such as those employed in construction and mining industries. Over time, exposure to silica particles causes scarring of the lungs, which can compromise breathing, and by promoting accumulative DNA damage, increases the risk of lung cancer [4, 5].

In contrast, in soft tissue regenerative therapy and bone augmentation prior to dental implant therapy, a few PRF matrices (~ 2, 3) are generally implanted a single time [1]. Accordingly, it is believed that neither the fibrosis nor accumulative DNA damage observed in workers chronically exposed to silica dust would likely occur in cells involved in regenerative dentistry. However, several more PRF matrices (~ 10) may be needed for one-time treatment of large tissue defects. In this case, it could not be ruled out that as an acute complication, inflammation is exerted or exacerbated at the implantation site or that as a delayed complication, increased DNA damage might accumulate locally in the cells around the implantation site, contributing to tumor formation.

Fortunately, there have been no reports of any obvious complications or adverse effects caused by silica microparticle (MP) contamination of PRF. Unless incorporated into the blood circulation, the potentially detrimental effects of silica, if any, may be limited. Nevertheless, given that the implantation site is typically inflamed during surgical procedures, the possible aggravation of pre-existing inflammation and/or delay of wound healing and tissue regeneration attributable to contaminant silica particles cannot be ruled out.

In this study, we sought to determine the stimulatory effects of silica MPs on the inflammatory system by examining the effects of sub-toxic doses of silica MPs on the proliferation and differentiation of human promyelocytic leukemia HL60 cells in vitro.

## Materials and methods

### Cell culture and morphology

For the purposes of this study, we used the human promyelocytic leukemia HL60 cells, human osteosarcoma-derived osteoblastic MG63 cells [6], and Balb/c mouse 3T3 cells [7], originally obtained from American Type Culture Collection (ATCC) (Gaithersburg, MD, USA) and stored in a laboratory liquid nitrogen tank. In our previous study [1], we used primary cultures of human periosteum-derived cells for cytotoxicity assays. Here, we used similar human osteogenic, but malignant MG63 cells as a reference. Although Balb/c cells are derived from mouse embryos, a preliminary study found that this cell line displays higher superoxide dismutase (SOD) activity than that of the other cells used here. Thus, we used Balb/c cells to test the involvement of SOD in silica cytotoxicity.

Fetal bovine serum (FBS), used to supplement culture media, was obtained from Gibco (Thermo Fisher Scientific, Waltham, MA, USA) and was heat-inactivated at 56 °C for 30 min prior to use. HL60 cells were cultured in RPMI 1640 medium (FUJIFILM Wako Pure Chemical Co., Osaka, Japan), supplemented with 1% heat-inactivated (hi)-FBS, in a humidified atmosphere containing 5% CO_2_ at 37 °C. MG63 and Balb/c cells were cultured under the same conditions in Dulbecco’s modified Eagle’s medium (DMEM) supplemented with 10% hi-FBS.

HL60 and the other cells (MG63 and Balb/c) were seeded into 40-mm culture dishes (TPP, Trasadingen, Switzerland) at a density of 7 and 4.5 × 10^4^ cells/dish, respectively, and cultured in the aforementioned growth media for 3 days. With regard to HL60 cell differentiation, 1 μM phorbol 12-myristate 13-acetate (PMA) (Adipogen Corporation, San Diego, CA, USA) and 1.2% dimethyl sulfoxide (DMSO) (FUJIFILM Wako Pure Chemical) were used for monocytic and granulocytic differentiation, respectively [8–11].

### Preparation of silica microparticle suspensions

Plastic vacuum tubes, the inner walls of which were coated with silica in the form of cerite (Neotube; Nipro, Otsu, Japan) were filled with 2 mL of RPMI supplemented with 1% hi-FBS or DMEM supplemented with 10% hi-FBS and vortexed to fully harvest the silica MPs from the tube. The resulting silica suspensions were stored at 4 °C until use (< 1 week). Prior to use, the silica suspensions were well vortexed and diluted with the same respective media [1].

### Scanning electron microscopy

Having carefully aspirated the medium from cultured cells to minimize cell detachment, cell morphology was examined using an inverted microscope (Eclipse Ti–U; Nikon, Tokyo, Japan) or a scanning electron microscope (TM-1000; Hitachi, Tokyo, Japan). Photomicrographs of living HL60 cells were obtained without fixation. With regard to sample preparation for scanning electron microscopy (SEM), the culture medium was carefully aspirated to minimize cell loss, and cultured cells tightly or weakly bound on plastic dishes were fixed with 2.5% neutralized glutaraldehyde, dehydrated, and freeze-dried, as described previously [12, 13]. The rim of the dish was removed, and thereafter, the fixed cells were subjected to magnetron sputtering and examined using SEM at an accelerating voltage of 15 kV.

### Determination of cell numbers

Following treatment, the media used for culturing HL60 cells was gently mixed 15 times with a dropper, and the floating and weakly bound cells suspended in the medium were counted using a MOXI Z automated cell counter (ORFLO, Ketchum, ID, USA). Moderately and tightly bound cells, which were resistant to initial gentle agitation, were treated with 0.05% w/v trypsin–0.53 mmol/L EDTA (FUJIFILM Wako Pure Chemical Co.) and detached by pipetting for adherent cell counts.

The MG63 and Balb/c cells tightly adhered to the bottom surface of the dish. The cells were not detached using gentle agitation. Thus, the culture media was aspirated without agitation, and the cells were enzymatically detached for cell counting, as described above.

### Cytochemical examination (acid phosphatase and non-specific esterase)

To determine the expression of acid phosphatases (ACP) and non-specific esterase (NSE), HL60 cells cultured in plastic dishes were fixed for 1 h with 10% neutralized formalin (FUJIFILM Wako Pure Chemical Co.) and stained for 30–60 min in a CO_2_ incubator using commercial kits (Muto Chemical Co., Tokyo, Japan), as described previously [14]. To enhance staining, we eliminated the counterstaining step and examined cells directly under an Eclipse 80i fluorescence microscope (Nikon, Tokyo, Japan) connected to a cooled VB-7000 CCD camera (Keyence, Osaka, Japan).

### Immunocytochemical fluorescence staining (CD11b)

Following incubation, HL60 cells were fixed for 1 h with 10% neutralized formalin (FUJIFILM Wako Pure Chemical Co.), washed twice with phosphate-buffered saline (PBS), and blocked for 30 min with 0.1% Block Ace (Sumitomo Dainippon Pharma Co. Ltd., Osaka, Japan) in 0.1% Tween 20-containing PBS. The samples were then treated for 45 min at room temperature (22–25 °C) with FITC-conjugated mouse monoclonal anti-CD11b antibodies (1:25 dilution; BioLegend, San Diego, CA, USA). For nuclear staining, the samples were further treated for 15 min with 1 μg/mL 4ʹ,6-diamidino-2-phenylindole (DAPI) (Dojin Chemical Lab., Kumamoto, Japan). Having washed with PBS, the samples were mounted using an antifade mounting medium (Vectashield®; Vector Laboratories, Burlingame, CA, USA), and examined under a fluorescence microscope (Nikon) connected to a cooled CCD camera (Keyence) [3].

### Determination of superoxide dismutase activity

Cultured cells were harvested using cell scrapers, washed with PBS, collected by centrifugation, and subsequently resuspended in PBS at higher densities (HL60: 2–10 × 10^6^/mL, MG63: 3–10 × 10^6^/mL, Balb/c: 1.8–10 × 10^6^/mL). Following sonication for 10 s, the cells were centrifuged at 10,000 × *g* for 15 min at 4 °C and the resulting supernatants were collected and stored at – 80 °C until use. Enzyme activity assays were performed using a SOD assay kit (Dojin Chemical Lab.) according to the manufacturer’s instructions.

### Statistical analysis

**Fig. 1:**
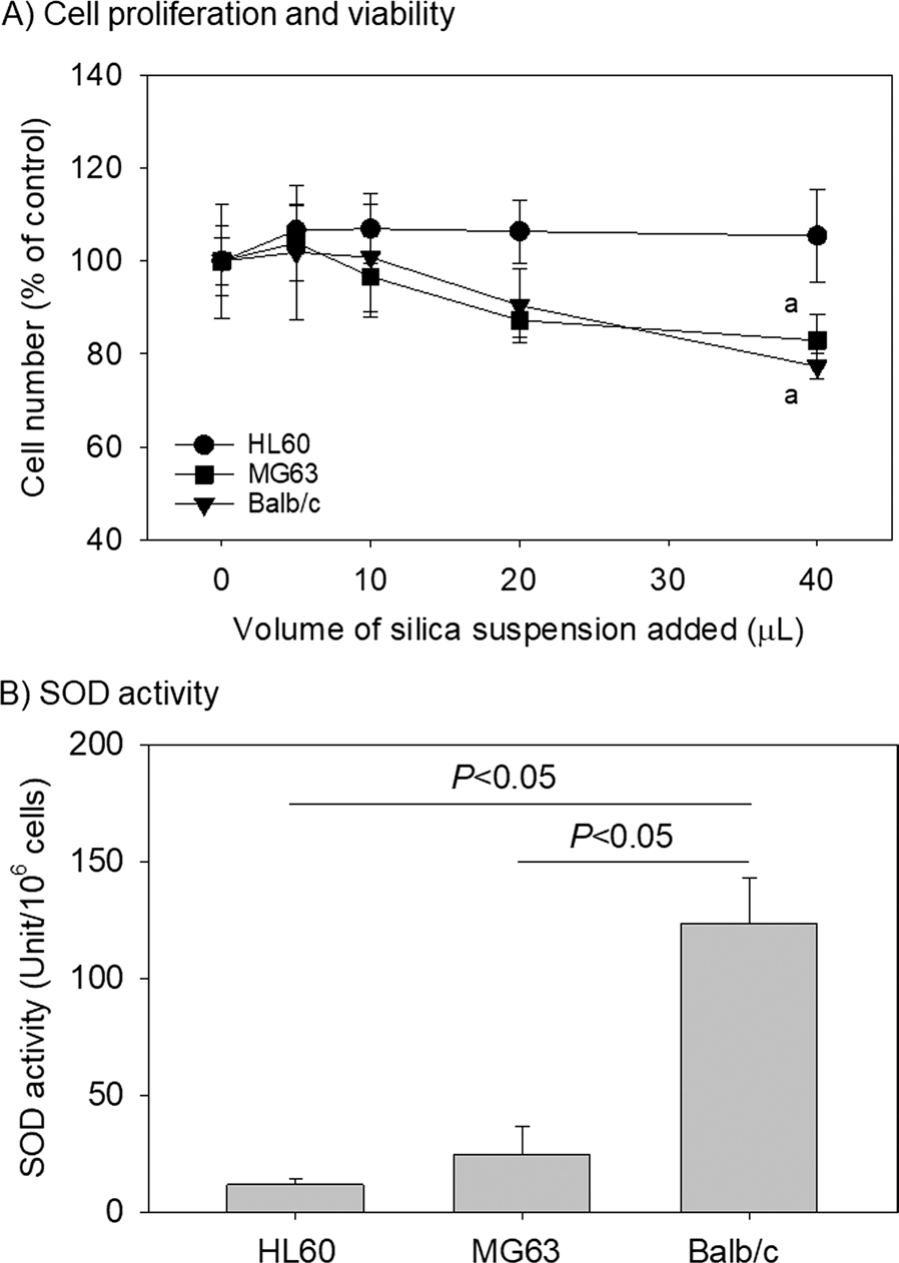
Dose-dependent effects of silica microparticles on the proliferation **A** and the superoxide dismutase (SOD) activity **B** of HL60, MG63, and Balb/c cells. *n* = 4 **A** and *n* = 5 **B** in independent cultures. The raw cell numbers at 100% represent 7.49 ± 9.84 × 10^5^ (HL60), 2.68 ± 0.16 × 10^5^ (MG63), and 3.09 ± 0.33 × 10^5^/dish (Balb/c), respectively. ^a^*P* < 0.05 represents a significant difference from the respective controls, to which no silica suspension was added

Data are expressed as the mean ± standard deviation of four or five independent cultures. For multi-group comparisons, when the data satisfied both normality (Shapiro–Wilk) and equal variance (Brown–Forsythe) assumptions, one-way analysis of variance (ANOVA) was performed, followed by a Bonferroni test (vs. control in Fig. 1A) or Tukey test (all pairwise comparisons in the remainder of the figures) as a post hoc test (SigmaPlot 13.0; Systat Software, Inc., San Jose, CA, USA). A *P*-value < 0.05 was considered to be indicative of a statistically significant difference.

## Results

### Effects of silica MPs on the proliferation of HL60, MG63, and Balb/c cells

Examination of the viability and proliferation of suspended and adherent cells demonstrated that no substantial cytotoxic effects of silica MPs at the examined doses were observed in HL60, MG63, or Balb/c cells regardless of the growing style (Fig. 1A). At 40 μL, however, we observed that silica MPs significantly suppressed (*P* < 0.05) the proliferation of adherent MG63 and Balb/c cells. Substantial differences were observed in terms of SOD activity. Specifically, Balb/c mouse cells were characterized by the highest SOD activity (*P* < 0.05), whereas human cells (HL60 and MG63) showed comparatively lower activity, irrespective of the culture type (Fig. 1B).

### Effects of silica MPs on the morphology of HL60 cells

**Fig. 2:**
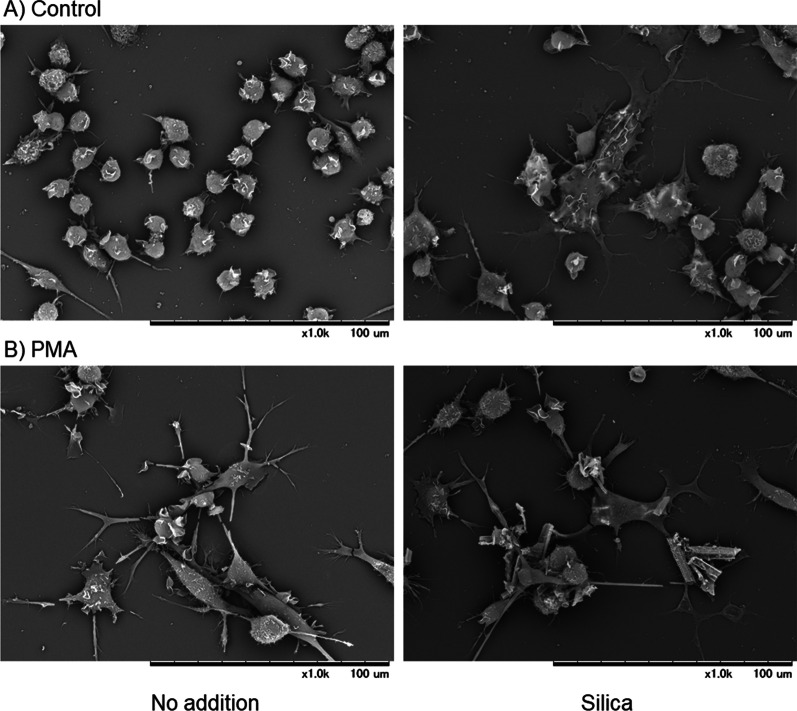
Effects of silica microparticles (MPs) on the surface microstructure of control and phorbol 12-myristate 13-acetate (PMA)-treated HL60 cells. The control cells **A** and those simultaneously treated with silica MPs for 3 days **B** were fixed and subjected to scanning electron microscopy examination. Similar data were obtained from two additional independent experiments (*n* = 3)

SEM examination (high magnification = 1000 ×) demonstrated that at a concentration of 1 μM PMA promoted the conversion of suspended and weakly bound HL60 cells to adherent cells with developed pseudopodia (Fig. 2B vs. A). When treated with 10 μL of silica MPs, a small percentage of control HL60 cells developed into elongated adherent cells (Fig. 2A). Moreover, we detected silica MPs attached to the plasma membrane, or incorporated into the cytoplasm of the control and PMA-induced cells.

**Fig. 3:**
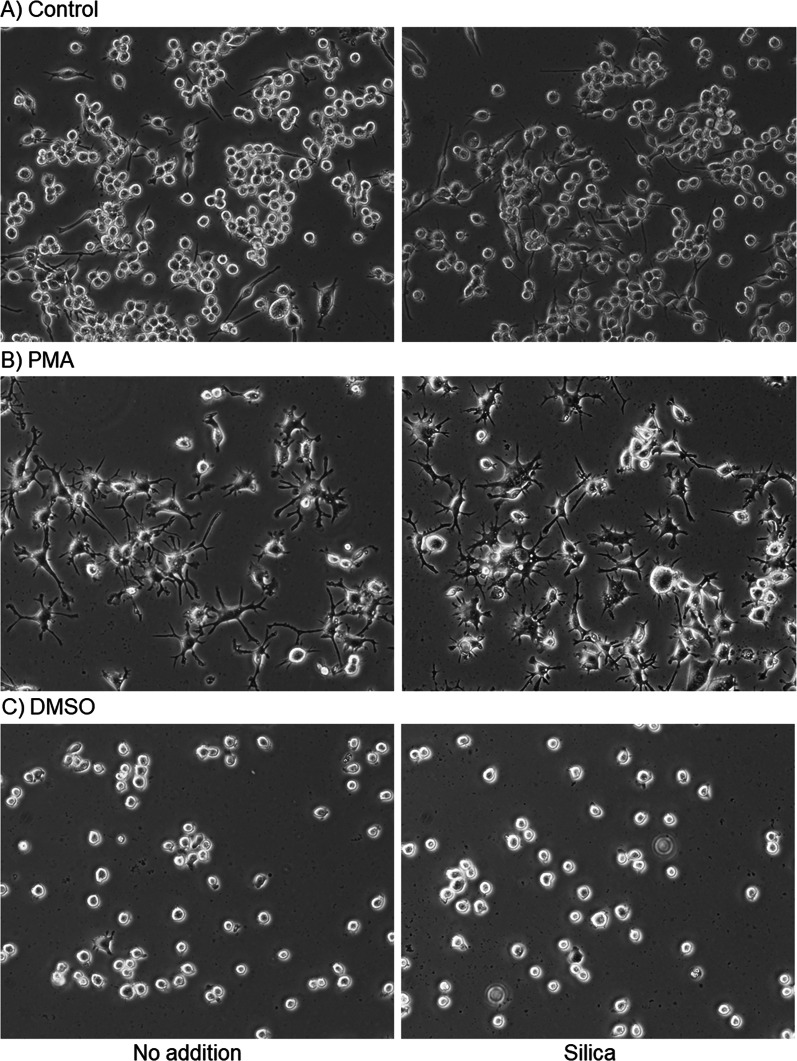
Effects of silica microparticles (MPs) on cell morphology in control **A** and phorbol 12-myristate 13-acetate (PMA)- **B** and dimethyl sulfoxide (DMSO)-treated HL60 cells (**C**). These cells were simultaneously treated with silica MPs for 3 days and examined microscopically without fixation. Similar data were obtained from two additional independent experiments (*n* = 3)

Microscopic examinations (low magnification) demonstrated that as observed in SEM findings, silica MPs (10 μL) induced minimal morphological changes associated with the transition of cells from a suspended to adherent form in the case of the control HL60 cells. In contrast, we detected no appreciable effects of MPs on PMA- or DMSO-induced cells. Microscopic examinations (low magnification =  ~ 200 ×) demonstrated that as observed in SEM findings, silica MPs (10 μL) induced minimal morphological changes associated with the transition of cells from a suspended to adherent form in the case of the control HL60 cells. In contrast, we detected no appreciable effects of MPs on either in PMA- and DMSO-induced cells (Fig. 3).

### Effects of silica MPs on the expression of differentiation markers of HL60 cells

**Fig. 4:**
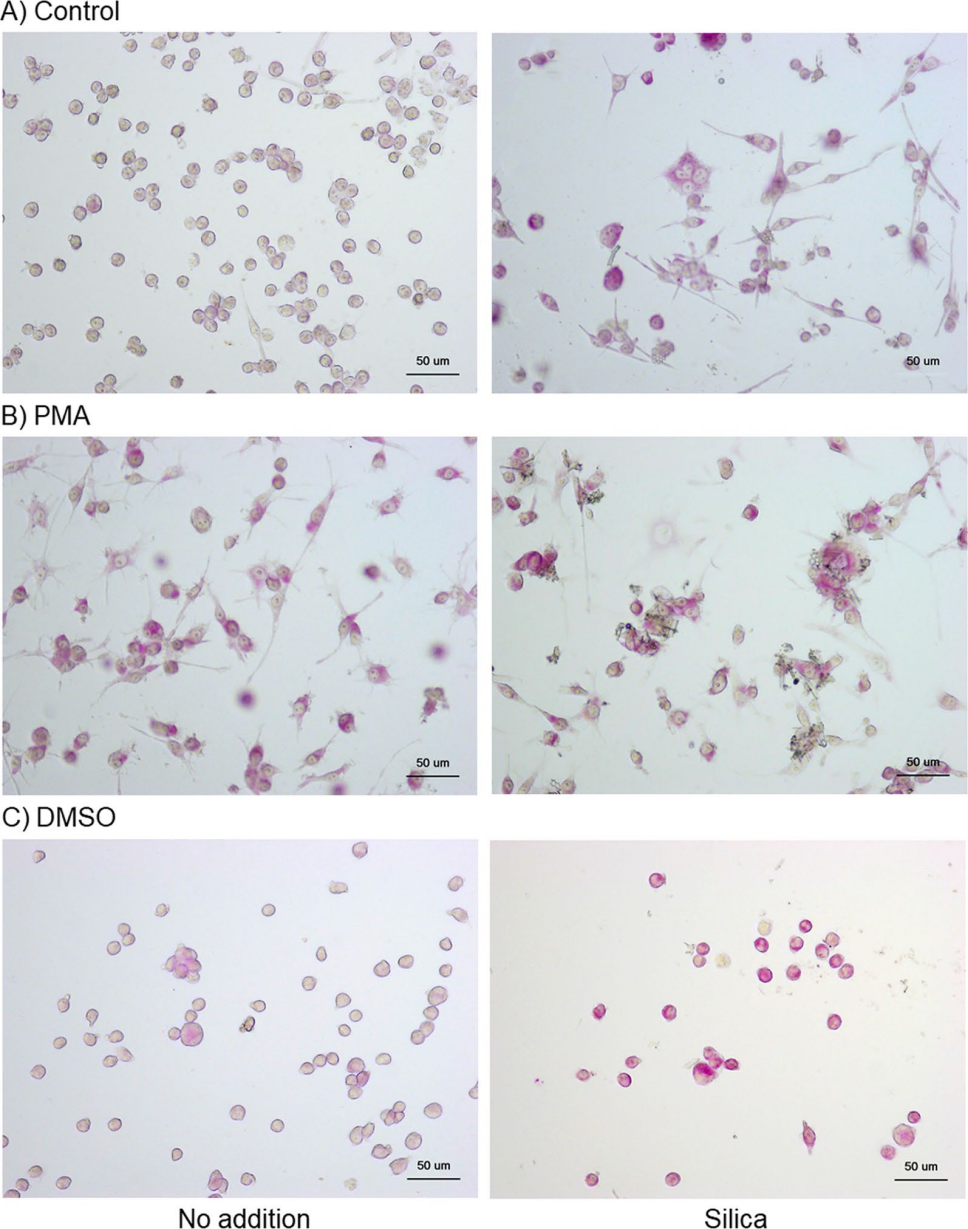
Effects of silica microparticles (MPs) on acid phosphatase (ACP) activity in control **A** and phorbol 12-myristate 13-acetate (PMA)- **B** and dimethyl sulfoxide (DMSO)-treated HL60 cells (**C**). These cells were simultaneously treated with silica MPs for 3 days. The cells were then fixed and subjected to cytochemical ACP staining. Similar data were obtained from two additional independent experiments (*n* = 3)

As previously observed [9], PMA and DMSO promoted high and low upregulation of ACP expression in the control HL60 cells, respectively (Fig. 4B, C vs. A). Furthermore, silica MPs (10 μL) substantially augmented ACP activity in both the control and differentiated HL60 cells.

**Fig. 5:**
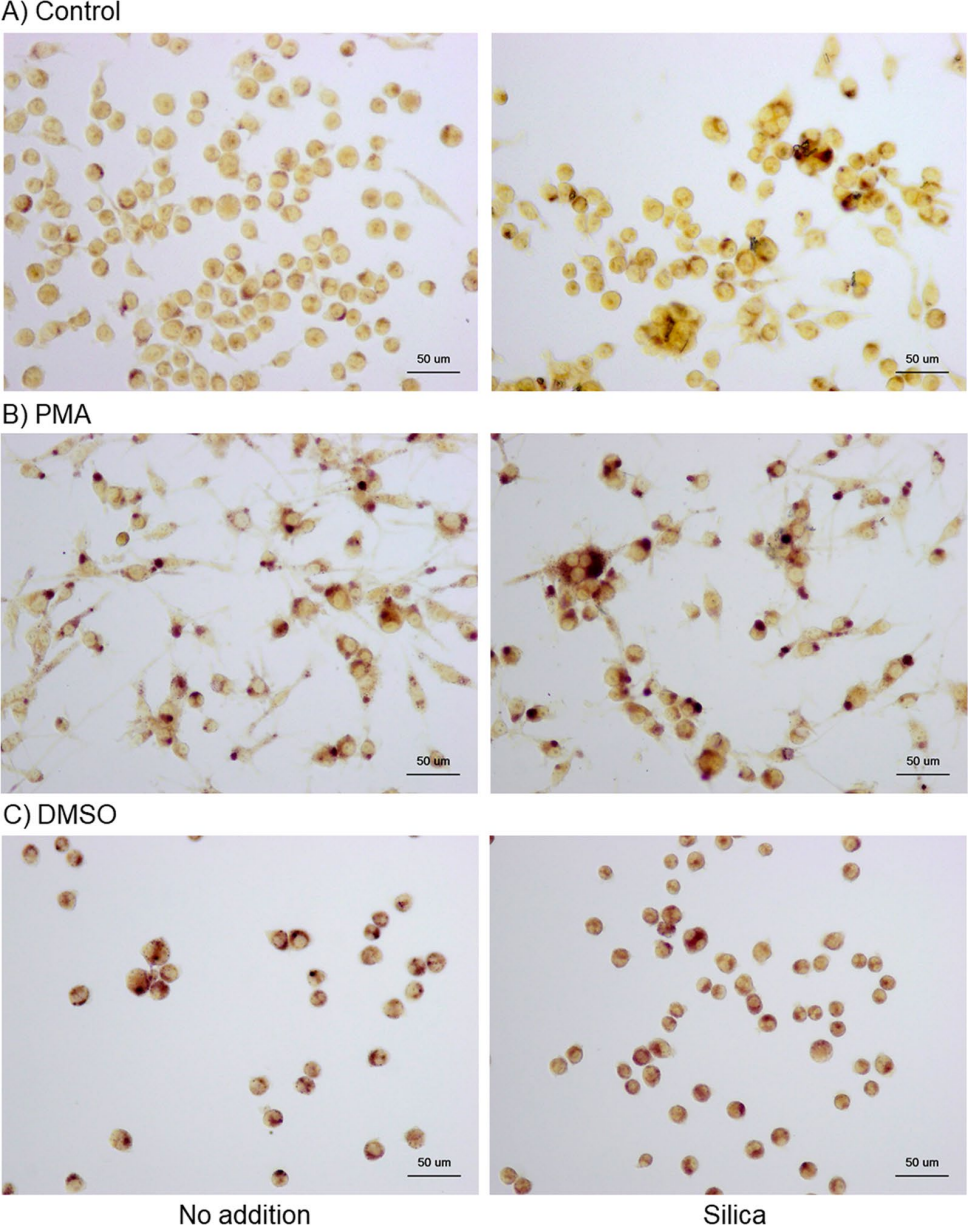
Effects of silica microparticles (MPs) on non-specific esterase (NSE) activity in control **A** and phorbol 12-myristate 13-acetate (PMA)- **B** and dimethyl sulfoxide (DMSO)-treated HL60 cells (**C**). These cells were simultaneously treated with silica MPs for 3 days. The cells were then fixed and subjected to cytochemical NSE staining. Similar data were obtained from two additional independent experiments (*n* = 3)

As described elsewhere [9], PMA and DMSO upregulated NSE expression in control HL60 cells (Fig. 5B, C vs. A). However, although silica MPs (10 μL) were observed to augment NSE activity in the control and PMA-induced HL60 cells, these particles appeared to have no significant effect on DMSO-treated HL60 cells.

**Fig. 6:**
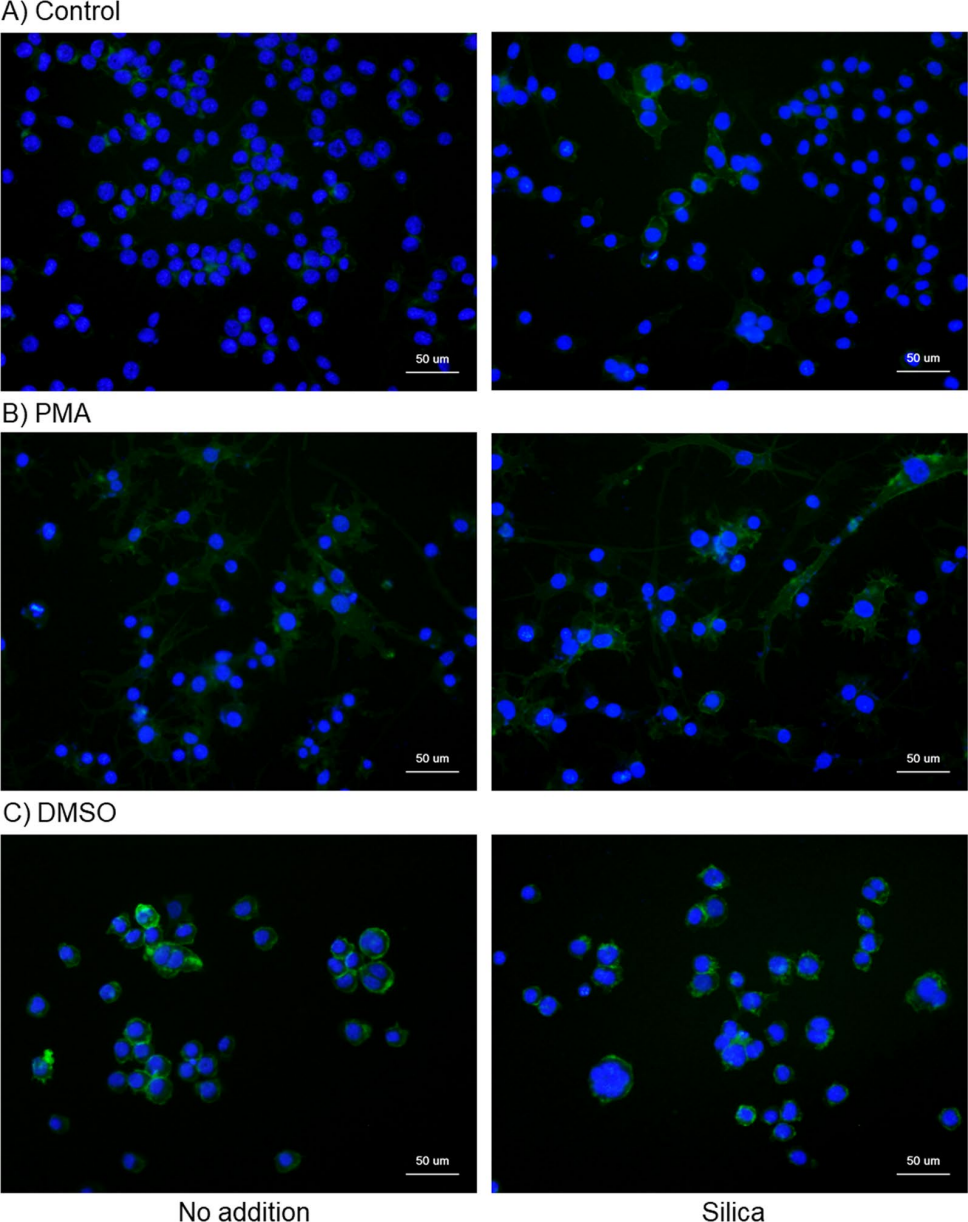
Effects of silica microparticles (MPs) on CD11b expression in control **A** and phorbol 12-myristate 13-acetate (PMA)- **B** and DMSO-treated HL60 cells (**C**). These cells were simultaneously treated with silica MPs for 3 days. The cells were then fixed and subjected to immunocytochemical CD11b staining followed by DAPI staining. Similar data were obtained from two additional independent experiments (*n* = 3). Green and blue signals indicate the presence of CD11b and nuclei, respectively

Consistent with previous observations [8], we found that PMA promoted the upregulated expression of CD11b in control HL60 cells, and similarly upregulated basal CD11b expression was detected in DMSO-treated cells (Fig. 6B, C vs. A). In a similar manner to the NSE activity, silica MPs (10 μL) augmented CD11b expression in both control and PMA-induced HL60 cells, although not significantly in the DMSO-treated HL60 cells.

### Effects of silica MPs on the proliferation and adhesion of control and differentiated HL60 cells

**Fig. 7:**
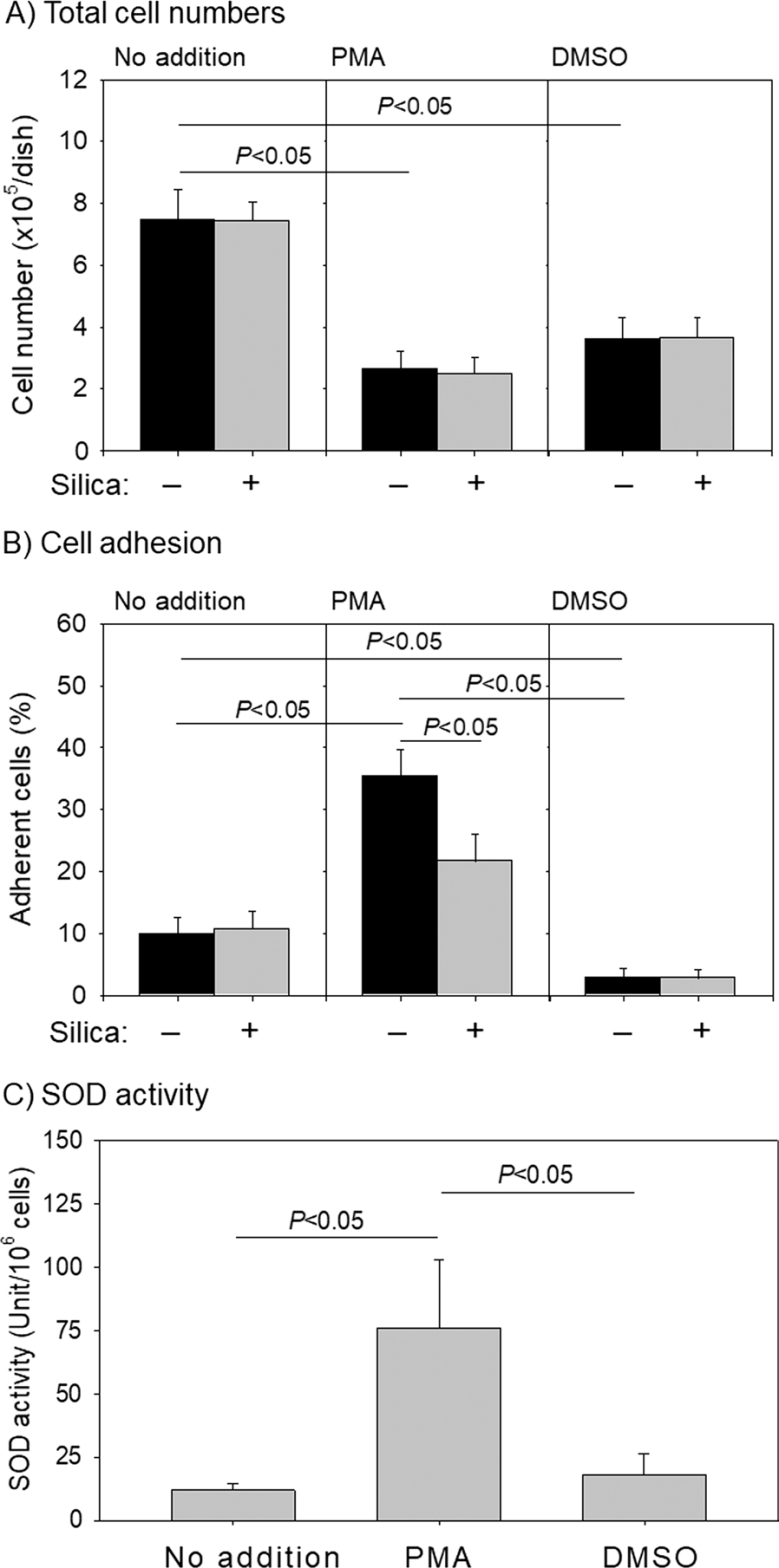
Effects of silica microparticles (MPs) on the proliferation, adhesion, and superoxide dismutase (SOD) activity of HL60 cells. After 3-day treatments, the suspended HL60 cells were collected and the adherent cells enzymatically detached for cell counting. **A** Total cell numbers are the sum of suspended and adherent cells *n* = 4. **B** The percentage of the adherent cells was calculated by dividing the adherent cell number by the total cell number *n* = 4. **C** SOD activity of the control and differentiated HL60 cells *n* = 5. *PMA* phorbol 12-myristate 13-acetate, *DMSO* dimethyl sulfoxide. *P* < 0.05 represent a significantly difference

The differentiation induction both by PMA and DMSO significantly (*P* < 0.05) suppressed cell proliferation, with PMA being observed to have a more pronounced effect in this regard (Fig. 7A). Contrastingly, as shown in Fig. 1, the proliferation of control HL60 cells was not suppressed by silica MPs (10 μL). Similarly, these MPs appeared to have no appreciable effect on the proliferation of either PMA- or DMSO-induced HL60 cells. Moreover, we observed that whereas silica MPs had a significant effect (*P* < 0.05) on the adhesion of PMA-induced HL60 cells, no similar effects were detected in the control or the DMSO-induced cells (Fig. 7B). Furthermore, although cell numbers were approximately equivalent, silica MPs were found to promote a significant reduction (*P* < 0.05) in the percentage of adherent PMA-induced HL60 cells. Nonetheless, compared with the control and DMSO-induced cells, we detected a significantly higher level (*P* < 0.05) of SOD activity in the PMA-induced HL60 cells (Fig. 7C).

## Discussion

In a preliminary study, we observed that higher doses of silica MPs apparently suppress the proliferation of HL60 cells. However, overall, the sensitivity of these cells to silica MPs appeared to be relatively lower than that of other assessed adherent cells. Taken together with the findings of the present study obtained using lower doses of these MPs, evidence would tend to indicate that direct contact may be prerequisite for silica MP-induced cytotoxic and/or differentiation-related effects on cultured cells in vitro.

It is generally believed that the cytotoxicity of silica is attributable to the activity of reactive oxygen species (ROS) generated on the surface of silica particles [15, 16]. ROS cause direct damage to the plasma membrane and DNA, thereby inducing cell death and tumor formation [17–19]. Given that the half-life of major ROS is relatively short (O^•−^_2_ and ^1^O_2_: 10^–6^ s, OH^•^: 10^–9^ s) [20], it is conjected that their detrimental effects are limited to cells that come into immediate contact with silica MPs. Theoretically, it is considered less probable that ROS would diffuse from the site of production and cause significant damage to neighboring or more distant cells within a few microseconds. Alternatively, it is plausible that silica MPs adhere to the plasma membrane [21] and may cause potential damage with sharp edges via mechanical means.

HL60 cells are considered a classical cell culture model for studying neutrophil functions and inflammatory cell responses [22]. To examine the effects of silica MPs on inflammation, we treated HL60 cells with comparatively low doses of these particles, with the findings indicating that silica MPs can stimulate an inflammatory response at the site of implantation. Although further clarification is necessary, it appears that, as observed in the case of cytotoxicity, silica-induced differentiation may be attributable to a cell contact-dependent, ROS-mediated mechanism.

It is also well established that neutrophils produce ROS primarily via NADPH oxidase, as part of the body’s immune response against invasive microorganisms [23]. In this regard, whereas in the undifferentiated state, HL60 cells lack an inherent ability to generate ROS in response to stimuli, having initially undergone PMA-promoted differentiation, these cells acquire ROS generation and antibiotic properties [24, 25]. In contrast, these properties tend to remain suppressed in DMSO-induced cells [25]. Consistent with these differential effects of different inducers, we found that PMA, although not DMSO, upregulated SOD activity, which is presumed to be indicative of a compensatory response to increased endogenous ROS generation in monocytic cells. In addition, we established that silica MPs enhance PMA-induced differentiation, either additively or synergistically. These findings would accordingly tend to imply that silica MPs have the capacity to influence the differentiation, not only of undifferentiated leukocytes but also monocytic cells at the early stages of differentiation, to induce and further exacerbate inflammation at the sites of implantation.

From a clinical perspective, it is believed that in soft tissues, such as gingival tissues, silica MPs delivered via PRF matrices are characterized by limited diffusion from the implantation site and undergo minimal entry into the circulatory system, unlike nanoparticles. Consequently, it appears that a major proportion of silica MPs that enter the body via this route remain at the implantation site. They promote damage to the connective tissue cells with which they come into direct contact with and induce inflammatory responses by promoting the differentiation of cells within the leukocyte lineage. In the case of bone augmentation, preceding surgical operations contribute to inducing inflammation; thus, it is conceivable that silica MPs exacerbate pre-existing inflammation, which has the effect of retarding bone regeneration. In both scenarios, the possible harmful effects would undoubtedly increase with the amount of implanted silica MPs [1]. Thus, in clinical cases that require multiple PRF matrices for the treatment of large connective tissue areas or bone defects, the use of silica-coated tubes for PRF preparation may increase the risk of severe complications. In such clinical applications, clinicians should be fully conscious of this risk.

Although compared with lung silicosis caused by chronic inhalation of silica dusts [26], the amount of silica MPs entering the body is considerably lower in the case of implantation and such implantation is generally infrequently repeated, we should, nevertheless, consider the similar possible risk of delayed toxicity. Further animal studies would thus seem necessary to assess the likelihood of this long-term effect. However, taking into consideration the prevailing 3R principles of animal experimentation (replacement, reduction, and refinement), it remains questionable as to whether we show persist down this particular avenue. Instead, unless biomedical advantages associated with the introduction of silica MP-contaminated PRF matrices can be demonstrated, we would recommend avoiding the use of silica-coated plastic blood collection tubes for the preparation of PRF in clinical settings.

## Conclusions

Collectively, the findings of this study indicate that silica MPs contaminating PRF preparations have the potential to acutely induce or exacerbate inflammation at the sites of implantation. Consequently, unless there are any perceived biomedical advantages associated with the introduction of these contaminated products, it would be prudent to find suitable alternatives to the use of silica-coated plastic blood collection tubes for the preparation of PRF.

## Data Availability

The data are available from the corresponding author on reasonable request.

## Abbreviations

MP: Microparticles
DMEM: Dulbecco’s modified Eagle medium
hi-FBS: Heat-inactivated fetal bovine serum
PBS: Phosphate-buffered saline
PMA: Phorbol 12-myristate 13-acetate
DMSO: Dimethyl sulfoxide
ACP: Acid phosphatase
NSE: Non-specific esterase
ROS: Reactive oxygen species
DAPI: 4’,6-Diamidino-2-phenylindole
